# Age is the main factor related to expiratory flow limitation during constant load exercise

**DOI:** 10.6061/clinics/2018/e439

**Published:** 2018-10-25

**Authors:** Patricia Rehder-Santos, Vinicius Minatel, Bruno Araújo Ribeiro, Richard Ducatti, Silvia Cristina Garcia de Moura-Tonello, Meliza Goi Roscani, Michel da Silva Reis, Ester Silva, Aparecida Maria Catai

**Affiliations:** I Laboratorio de Fisioterapia Cardiovascular, Nucleo de Pesquisas em Exercicio Fisico, Departamento de Fisioterapia, Universidade Federal de Sao Carlos, Sao Carlos, SP, BR; II Departamento de Medicina, Universidade Federal de Sao Carlos, Sao Carlos, SP, BR; III Grupo de Pesquisa em Avaliacao e Reabilitacao Cardiorrespiratoria (GECARE), Departamento Fisioterapia, Faculdade de Medicina, Universidade Federal do Rio de Janeiro, Rio de Janeiro, RJ, BR

**Keywords:** Pulmonary System, Exercise, Aging, Healthy Individuals, Physical Activity

## Abstract

**OBJECTIVE:**

The objective of this study was to investigate the interaction among the determinants of expiratory flow limitation (EFL), peak oxygen uptake (VO_2_peak), dysanapsis ratio (DR) and age during cycling at different intensities in young and middle-aged men.

**METHODS:**

Twenty-two (11 young and 11 middle-aged) men were assessed. Pulmonary function tests (DR), cardiopulmonary exercise tests (VO_2_peak) and two constant load tests (CLTs) at 75% (moderate intensity) and 125% (high intensity) of the gas exchange threshold were performed to assess EFL. EFL was classified using the percentage of EFL determined from both CLTs (mild: 5%-30%, moderate: 30%-50%, severe: >50%).

**RESULTS:**

Only the middle-aged group displayed EFL at both exercise intensities (*p*<0.05). However, the number of participants with EFL and the percentage of EFL were only associated with age during high-intensity exercise.

**CONCLUSIONS:**

There was no interaction between the determinants. However, age was the only factor that was related to the presence of EFL during exercise in the age groups studied.

## INTRODUCTION

During exercise, the pulmonary system works in coordination with the cardiovascular and musculoskeletal systems to promote adequate alveolar ventilation, efficient gas exchange with low energy expenditure, and good performance 1,2. Nevertheless, during exercise, healthy individuals, regardless of their physical fitness, can experience expiratory flow limitation (EFL), which is characterized by the inability to increase expiratory flow even with increased transpulmonary pressure 3.

EFL can be observed when the exercise expiratory flow-volume loop (EFVL) reaches or exceeds the maximal flow-volume loop (MFVL) 3. The presence of EFL is an important clinical indication because it can exert significant effects on ventilatory capacity due to the approximation of maximal expiratory flow, which leads to changes in respiratory mechanics, ventilation control, dyspnea and exercise intolerance 4. An increasing number of studies in recent years have attempted to determine the main predictors of the onset of EFL. This would enable early intervention in patients where EFL is accompanied by clinically relevant changes, such as pulmonary hyperinflation 5, increased work during breathing 6,7 and exercise-induced arterial hypoxemia 8. The main determinants of EFL are aerobic capacity, age and structural changes.

Aerobic power determined by peak or maximal oxygen uptake during exercise (VO_2_peak or max) has been considered one of the main determinants of EFL 6,9. Several studies have suggested that endurance-trained men and women have higher EFL values than individuals who have not undergone this type of training because it is assumed that well-trained individuals have a higher ventilatory demand, exceeding the pulmonary thresholds and triggering EFL 6,9. However, aerobic power does not explain the presence of EFL in individuals with the same functional capacity 10.

Therefore, there has been renewed interest in the concept of dysanapsis, according to which an individual with larger lungs does not necessarily have larger airways than individuals with smaller lungs 11. This concept gave rise to the dysanapsis ratio (DR), which indirectly and noninvasively measures the relationship between lung size and airway size and has important clinical relevance 11.

The DR has already been used to determine whether EFL is related to anatomical factors or aerobic power 3,10. Dominelli et al. 10 studied healthy women and found that lower DRs are related to the prevalence of EFL in this population. Additionally, Smith et al. 3 assessed young men and women with the same level of fitness and concluded that the DR is a predictor of EFL. However, no studies have compared the DR in different age ranges among individuals of the same sex, as proposed in this study.

Another factor that can be considered for determining EFL is age given the morphological and functional changes that occur as a result of aging, such as reduced pulmonary function and compliance 4,12. Nevertheless, no studies have assessed the interaction among these factors in relation to EFL in individuals of the same sex but of different ages (young versus middle-aged) and at different exercise intensities [75% (moderate intensity) and 125% (high intensity) relative to the gas exchange threshold (GET)]. Evaluating middle-aged people is important because this population represents subjects who are at the peak of productivity and can be affected by chronic cardiorespiratory diseases. Determining factors that can predict exercise limitation early on, preceding the onset of clinical symptoms, can help health professionals choose tools to reduce these changes.

Therefore, the purpose of the present study was to evaluate whether EFL in healthy men from two different age groups is related to a decrease in aerobic power (VO_2_peak), pulmonary structural changes (DR) and/or aging. Thus, the aim of this study was to investigate the interaction between EFL determinants (VO_2_peak, DR and age) during exercise at different constant intensities performed using a cycle ergometer (75% of the GET and 125% of the GET) and in different age groups (young and middle-aged).

## MATERIALS AND METHODS

### Design

This is a prospective, observational, descriptive and cross-sectional study.

### Subjects

The study included 22 apparently healthy men: 11 in the young group (YG: 24±3 years) and 11 the middle-aged group (MAG: 48±4 years). The subjects were recruited by electronic and printed media, and all of them were classified as physically active according to the American College of Sports Medicine (ACSM) 13 (minimum of 150 min of weekly physical activity, as reported from memory). The inclusion criteria were as follows: no use of prescription medications, illicit drug use and/or smoking; no diagnosis of cardiorespiratory or metabolic disease; absence of ischemic and conduction alterations on ECG at rest or during the clinical exercise test; body mass index (BMI)<30 kg/m^2^; and absence of respiratory muscle weakness [maximal inspiratory pressure (MIP<60% predicted) 14] and alterations in the pulmonary function test (PFT). The present study was conducted in accordance with the Helsinki Declaration. This study was approved by the Human Research Ethics Committee at the Federal University of São Carlos (UFSCar) (n°. 403.589). All subjects signed an informed consent form.

## METHODS

The tests and experimental procedures were conducted at the UFSCar Cardiovascular Physical Therapy Laboratory in a climate-controlled room (temperature 21-24°C; relative humidity 40-60%). The tests took place in the afternoon, taking into account the influence of the circadian cycle on the test results. The subjects were instructed to rest well the night before, wear comfortable clothes and shoes, and avoid alcoholic or stimulating beverages and strenuous exercise the day before the assessment.

Before starting the experimental procedures, all subjects underwent an assessment of medical history based on their memory, physical examination, conventional 12-lead rest ECG and a clinical exercise test on a treadmill (Ellestad protocol) under the supervision of a cardiologist. These tests were performed to determine eligibility. The subjects included in the present study were familiarized with the equipment and respiratory maneuvers to reduce anxiety and avoid the interference of learning effects on the results of the study. Moreover, on the day of the tests, the health condition of the subjects was assessed prior to starting the experiment.

Data collection took place on two separate days, with a minimum interval of 48 h and a maximum interval of 1 week. On the first day, respiratory muscle strength was tested, followed by administration of the cardiopulmonary exercise test (CPET). On the second day, the subjects underwent a PFT before and after two constant load tests (CLTs). During these tests, the arterial pressure, heart rate (before, during exercise at each intensity and after the experiment) and ECG traces were continuously monitored. Ventilatory and metabolic variables were captured breath by breath during the entire period of administration of the CPET and CLT, using a gas exchange analysis system (ULTIMA/Breeze Suite 7.2. MedGraphics Breeze, St. Paul, MN, USA).

Maximal expiratory pressure (MEP) and MIP were measured according to the American Thoracic Society (ATS)/European Respiratory Society (ERS) 15 guidelines, and the values predicted for the Brazilian population were calculated according to Neder et al. 16. After undergoing the respiratory muscle assessment, each subject performed the CPET. The CPET was used to assess aerobic power and determine the GET 17,18. A cycle ergometer with electromagnetic braking was used for this test (CORIVAL V3, Lode BV, Groningen, Netherlands). The protocol began with 1 min of rest with the subject seated on the cycle ergometer, followed by 3 min of load-free exercise and then exercise with incremental increases in load. The power was calculated for each subject according to values established using the formula by Wasserman et al. 2. The participants were instructed to maintain a cadence of 60 rpm, and the test lasted 8-12 min 17. The interruption criteria for this assessment were in accordance with Balady et al. 17.

To determine the GET, we used the mean of the assessments of three independent assessors, considering a difference less than or equal to 2% among each value 19. The mean VO_2_ value obtained in the last 30 seconds of CPET was considered the VO_2_peak 17. The following variables were assessed in relation to the GET and exercise peak: VO_2_, carbon dioxide production (VCO_2_), respiratory exchange ratio (RER), lung ventilation (VE), oxygen uptake efficiency slope (OUES), and minute ventilation-carbon dioxide production slope (VE/VCO_2_ slope) 17.

PFTs were conducted according to the standards of the ATS/ERS 15 on the day of the initial assessment to avoid including subjects with any lung disorders and were also conducted before and after each CLT 14 to obtain the necessary variables to determine the MFVL and compare the variables obtained during the CLTs, as well as to ensure the quality of the collected data and identify the respiratory pattern of the subjects. After the CLT, the PFT was used to exclude the presence of exercise-induced bronchodilation 9,20.

The analyzed variables were as follows: forced vital capacity (FVC), ratio of forced expiratory volume in 1 second (FEV1) to FVC (FEV1/FVC), inspiratory capacity (IC), expiratory reserve volume (ERV), and maximal voluntary ventilation (MVV). The predicted values for the Brazilian population were calculated according to Pereira 21. In addition, the DR was calculated using a noninvasive method (DR=forced expiratory flow at 50% of FVC / [FVC × (-0.056 × age + 6.3038)] 3.

Two CLTs were conducted on the cycle ergometer to assess EFL using the EFVL method during exercise. The load corresponding to the GET [considering power (W)] during the CPET was used to calculate the intensity used in the CLTs. Moderate intensity corresponded to 75%, while high intensity corresponded to 125% of the GET 22. The test consisted of 1 min of rest, followed by 4 min of load-free warm-up and 10 min of exercise with the load set for the test (75% or 125% of the GET). In the last 4 min of each CLT, EFVL was plotted. After the load was removed, 6 min of load-free active recovery and 1 min of passive recovery were allowed.

**Figure 1:**
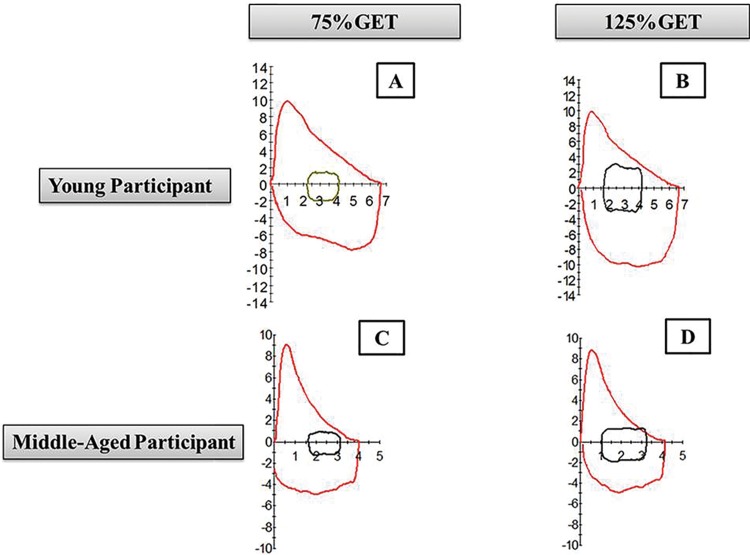
Illustration of the expiratory flow-volume loop (EFVL), plotted within the maximal flow-volume loop (MFVL) for each exercise intensity. Young participants exercising at loads of 75% of the gas exchange threshold (GET) (A) and 125% of the GET (B); middle-aged participants exercising at loads of 75% of the GET (C) and 125% of the GET (D) and showing expiratory flow limitation (EFL).

The EFL was assessed by comparing the EFVLs during the IC maneuvers, and the MFVL was measured before each CLT 7. Three IC maneuvers were performed with 1-min intervals between them, and they were used to correctly position the EFVLs in the MFVL 22 (Figure 1). For the analysis, three EFVLs were selected for each intensity, and the most representative value was used. The subject had a 30-min rest between tests at each load 22, and the order of the exercise intensities was randomized.

The degree of EFL was defined as the percentage of EFVLs exceeding the MFVL. A minimum of 5% tidal volume overlap was required for subjects to be considered as having EFL 23. The analyzed variables were as follows: number of subjects with or without EFL in each group and at each intensity, VO_2_peak, IC, maximal expiratory and inspiratory flow (FEFmax and FIFmax, respectively) and percentage of EFL 21. The EFL classification was used in accordance with Johnson et al. 7; percentages below 30% were considered mild, 30% to 50% moderate and above 50% severe.

### Statistical analysis

First, the sample size was calculated using G*Power 3.1.3 software and defined by the analysis of the percentage of EFL at 125% of the GET, determined using unpaired Student's t test during the pilot test (YG=4; MAG=4) with a power of 95%. A sample calculation was performed in a previous study conducted by our group, which identified the presence of EFL in a middle-aged group 24. The EFL percentage was used for all calculations, with 4 subjects for each group.

For data normality and homogeneity distribution analyses, we used the Shapiro-Wilk and Levene's tests. The data are shown as the mean±standard deviation (SD) or median (minimum-maximum). Afterwards, the non-parametric Student's t test or the Mann-Whitney test were used to compare the groups for anthropometric variables and for respiratory muscle strength test, PFT and the cardiopulmonary test variables. Two-way ANOVA was used to analyze the effect of age and exercise intensity on the CLT variables. Multiple stepwise regression was used to analyze the interaction between the VO_2_peak, DR and age. The data were processed using SPSS 17.0 software. The significance level was set at *p*<0.05.

## RESULTS

Table 1 presents the anthropometric and respiratory muscle strength data and the main PFT indices for each group. The absolute values of PFT indices were lower in the MAG, and the percentage of the predicted IC was higher in the MAG than in the YG (*p*<0.05). However, no differences were observed between the variables analyzed for the GET and at the peak of exercise, in the power attained (increment and peak) or in the ventilatory and metabolic variables (*p*>0.05) (Table 2). Additionally, functional aerobic classification was carried out according to the ACSM 13; the YG had 8 subjects classified as low and 3 as regular, while the MAG had 9 subjects classified as regular and 2 as good. Notably, signs or symptoms of exercise intolerance were not observed in either group.

Table 3 presents the results related to the influence of age and exercise intensity during the CLTs. Compared to the YG, the MAG displayed EFL at both intensities (*p*<0.001). Moreover, the VO_2_peak, EFL, power, FEFmax and FIFmax were only higher with high-intensity exercise (125% of the GET) (*p*<0.05). However, IC did not show any influence of age or intensity and this did not change between the conditions (*p*=0.266 and *p*=0.674, respectively).

**Figure 2:**
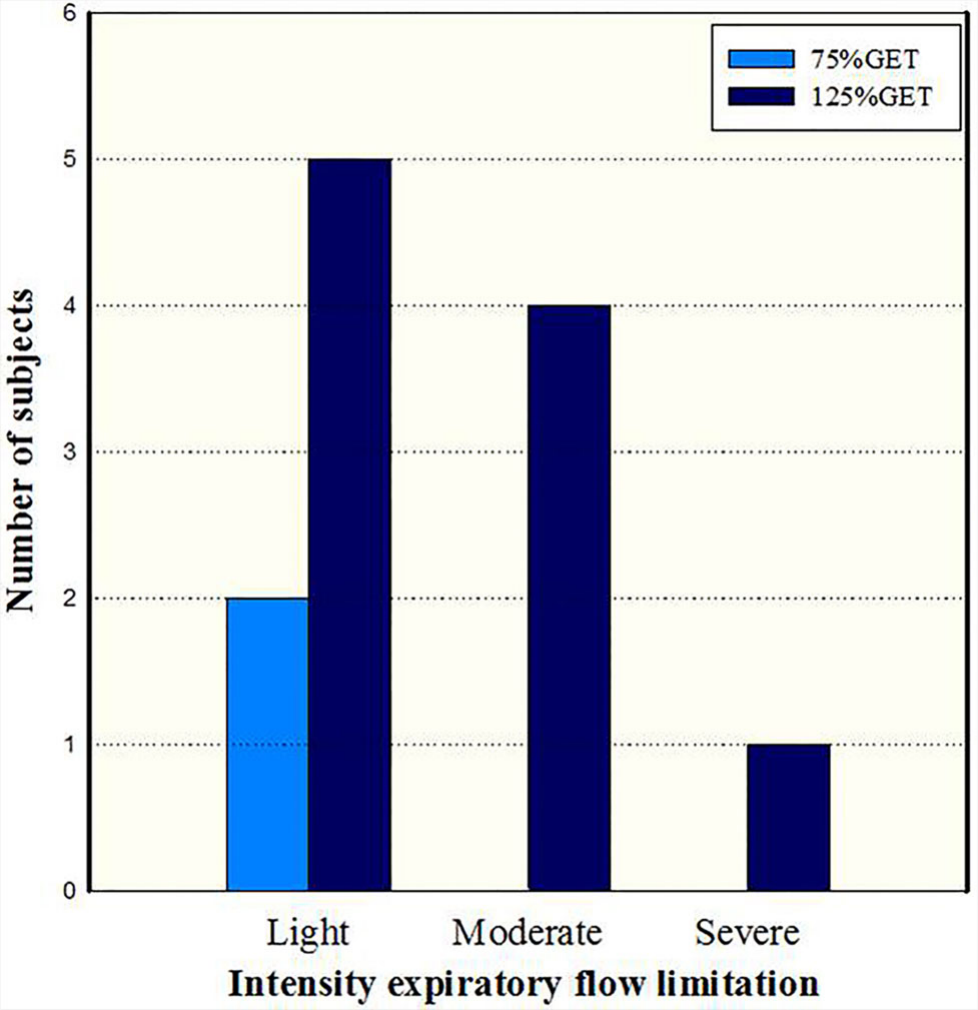
Expiratory flow limitation (EFL) during exercise on a cycle ergometer in the middle-aged group during moderate (75% of the gas exchange threshold (GET)) and high-intensity (125% of the GET) exercise [classification based on Johnson et al. 8].

Regarding the classification of EFL at different exercise intensities, only results for the MAG are shown in Figure 2 as no subjects from the YG displayed EFL. The main results of our study demonstrate an interaction between the determinants of EFL (VO_2_peak, DR and age) during moderate (75% of the GET) and high-intensity (125% of the GET) exercise. No interaction was detected between these determinants and the presence of EFL at 75% of the GET. However, there was an interaction between the age and the number of participants who displayed EFL (r=0.82; r^2^=0.62; β=0.82; *p*=<0.001) and the percentage of EFL (r=0.67; r^2^=0.42; β=0.67; *p*=0.001) with high-intensity exercise.

## DISCUSSION

The main findings of the present study were as follows: a) there is no interaction among the determinants VO_2_peak, DR and age in healthy young and middle-aged active men and b) age is related to the presence and percentage of EFL during constant load exercise on a cycle ergometer.

The fact that the subjects in the MAG displayed more EFL and were more active than those in the YG, despite the lack of difference in the DR between the groups, shows that age contributed more to EFL during physical exercise than the other factors. This result demonstrates the need to incorporate prevention activities before old age, as severe EFL can limit the practice of physical exercise.

Furthermore, it is worth noting that most studies on this topic take into account only the extreme age ranges and do not consider what happens in the range studied in this research (middle-aged versus young). The analysis of the interactions among the variables showed that age was the only factor that interacted with EFL, while the DR and VO_2_peak had no relationship with the number of participants who displayed EFL or with the percentage of EFL, showing that these variables could not explain the onset of EFL in the MAG. These data show that with age, there is an increase in both the occurrence and percentage of EFL. These findings emphasize the need for studying the cause of EFL in middle-aged individuals because the main determinants used in the literature (DR and VO_2_peak) fail to explain the results of this study, thus limiting the early identification of cases where the EFL is classified as moderate or severe.

The observation that the DR was not related to the presence of EFL during exercise in either group is inconsistent with the evidence presented by Dominelli et al. 10 regarding the use of the DR as a major determinant of EFL. Notably, these authors studied healthy young women, for whom lower DR values resulted in a higher prevalence of EFL. In addition, Smith et al. 3 showed that the DR was a predictor of EFL during dynamic exercise evaluated in healthy, active, young people of both sexes. The differences presented by our study can be explained by the addition of an active, middle-aged group.

In the analysis of data from CPETs, we observed no differences between groups in relation to the rate of incremental change and to the variables power attained, VO_2_ and VCO_2_ (at the GET and at the peak of exercise). These data show once again that subjects in the MAG were transitioning from balanced cardiorespiratory indices to age-related cardiorespiratory disorders, thereby demonstrating the onset of EFL in this age group. In addition, there were no significant differences between the groups in the VE/VCO_2_ slope or OUES (Table 2); thus, it is not possible to conclude whether EFL is being triggered by a reduction in respiratory efficiency. Therefore, our results suggest that EFL in the MAG may have been due to age-related structural changes that were not identified by the assessments conducted in this study. We emphasize that our study stands out as it evaluates subjects that are active but not athletes, which is typical of the lifestyle of most of the population. The use of these subjects could explain the absence of an interaction between aerobic performance (VO_2_peak) and EFL, as the two groups did not differ with respect to the VO_2_peak as measured through the CPET (Table 2). This finding may also be related to the age groups chosen for the study, as the changes in structure and respiratory mechanics due to aging, which should have been evident when comparing the YG with the MAG, were not sufficient to identify significant differences in the variables studied. However, differences between the groups were observed concerning aerobic functional classifications, according to the ACSM guidelines 13; the MAG had a better classification than the YG.

Regarding EFL distribution in the different intensities in the MAG, only two subjects displayed mild EFL at 75% of the GET and at 125% of the GET, whereas one subject did not display EFL, five displayed mild EFL, four displayed moderate EFL, and one displayed severe EFL (Figure 2). In contrast, none of the subjects in the YG displayed EFL at either exercise intensity. The presence of EFL during exercise in the MAG can be explained by the decrease in elasticity and the thickening of the thoracic walls as a result of aging 25. These changes in elastic mechanisms can lead to increased intrathoracic pressure, reducing the internal pressure of the airways due to increased ventilatory demand during exercise, thus worsening dynamic compression of the airways and reducing maximal expiratory flow 25.

In relation to the effect of age during constant load exercise, the percentage of EFL was the only affected variable (Table 3). Furthermore, exercise intensity led to higher values for the VO_2_peak, percentage of EFL, FEFmax and FIFmax. These results had been expected given that with an increase in ventilatory demand there is an increase in maximal inspiratory and expiratory flow to maintain adequate ventilation for exercise at different intensities.

The anthropometric characteristics (body mass, height and BMI), respiratory muscle strength and results of PFTs show that the groups were within the range of values predicted for their age, which confirms that the inclusion criteria were fulfilled. The YG also had higher values for the absolute indices (FVC, FEV1, ERV and MVV) and for the FEV1/FVC ratio than the MAG, and this result was expected given the effects of aging 24. Moreover, these results are supported by the fact that no differences were found in these indices between the percentage of values obtained and the values predicted for each age group.

However, such findings should be considered with caution because healthy men aged 20 to 40 years have a good expiratory reserve, enabling increased ventilatory demand even during peak exercise 1. Additionally, when the ERV is decreased, as observed in the present study, the values obtained for the MFVL originating from FVC maneuvers in the MAG decrease (Figure 1) 1. Therefore, the presence of EFL in the MAG can be explained by the consequent decrease in MFVL in this group (Figure 1) 1. This finding can be compared to the results of a review by Sheel & Guenetti 1, which showed that the increased prevalence of EFL in women compared to that of EFL in men in the same age group is due to the lower lung volume of women resulting from anatomical and structural differences between the sexes. Thus, the responses in the MAG in our study seem to be similar to those in the women studied in the above mentioned review.

Nevertheless, the presence of EFL in the MAG even without intrinsic changes demonstrated that EFL may be the first indication of the onset of respiratory symptoms such as dyspnea 26 or functional changes in the elderly, such as a decrease in ventilatory efficiency 27 and pulmonary function 28. These changes in the respiratory system may interfere with the health and well-being of the subjects, as outlined by Waterer et al. 29, who studied the elderly of both sexes and concluded that EFL in this population is underdiagnosed and can be an early indication for diseases of the respiratory tract.

The main limitation of this study is the absence of an invasive method to evaluate the DR, lung volumes and pulmonary capacities, as reported by Dominelli et al. 30. Such methods could help us identify the structural changes that occur with aging in the pulmonary system, which could be expressed by the DR 30. However, the DR obtained using a noninvasive measure was sufficient to identify the relationship between EFL and changes in airway and lung structure. Moreover, noninvasive measurement of pulmonary volumes and capacities is the cheapest way to evaluate a large population and can be used in clinical and rehabilitation assessments. This is important as it suggests that EFL during aging precedes the onset of clinical symptoms, such as intolerance to high-intensity exercise, dyspnea and a significant reduction in aerobic functional capacity, at least in the age groups studied. In addition to EFL, there was no interaction with anatomical changes, such as the size of the airways and lung area as evaluated by the DR.

Another limitation of our study was the similarities between groups, as we did not expect that the evaluated groups would be similar with respect to pulmonary function, oxygen consumption, or other factors. As demonstrated by Sheel and Guenette et al. 1, in healthy young men (aged 18-40 years), there is a large reserve for increasing ventilation even at peak exercise. However, after 40 years of age, the respiratory variables begin to decrease. The sample size was also a limitation. EFL was considered an outcome variable to calculate the sample size; however, the calculated number of subjects was not sufficient to identify the differences in DR and VO_2_peak. These limitations may have affected the findings of this study.

The use of MFVL to determine EFL has also been questioned 7,21; however, it is noteworthy that in the present study, we only considered EFL percentages higher than 5% 22 as a criterion for data analysis.

The results of this study highlight the importance of careful and early EFL evaluation in middle-aged subjects to prevent and minimize complications in the cardiorespiratory system resulting from aging and thus improve the quality of life and enable the maintenance of physical activities for a long time.

In conclusion, age was the only factor that interacted with EFL in the MAG, and the greater the intensity of dynamic exercise, the greater the percentage of EFL displayed by these individuals. Furthermore, the DR and VO_2_peak indices were not sufficient to determine EFL in healthy young and middle-aged active men.

## AUTHOR CONTRIBUTIONS

Rehder-Santos P and Catai AM conceived of the hypothesis and design of the study, contributed to the manuscript preparation, reviewed all versions of the manuscript and took responsibility for all aspects of the reliability and freedom from bias of the data presented. Rehder-Santos P, Minatel V, Ribeiro BA, Ducatti R and Moura-Tonello SC contributed to the study, screened the volunteers and performed the experiments. Roscani MG carried out clinical evaluations. Rehder-Santos P, Minatel V, Reis MS, Silva E and Catai AM performed the data analyses. Rehder-Santos P and Minatel V performed the statistical analyses. All authors participated in data interpretation and critical review of the manuscript and approved its final version.

## Figures and Tables

**Table 1:** Characterization of the study subjects, including age, anthropometric data, respiratory muscle strength, pulmonary function test parameters and dysanapsis ratio.

	YG (n=11)			MAG (n=11)		*p* value
Age (years)	24±3			48±4		**<0.001**
**Anthropometric Data**						
Mass (kg)	81±10			80±13		0.79
Height (cm)	177±6			172±8		0.09
BMI (kg/m^2^)	26±2			27±3		0.30
**Respiratory Muscle Strength**						
MEP (cmH2O)	173±21			183±54		0.62
% Predicted	118±13			145±43		0.08
MIP (cmH2O)	153±11			143±18		0.12
% Predicted	112±8			127±23		0.13

Pulmonary Function Test	Absolute Value			% Predicted		
	YG (n=11)	MAG (n=11)	*p* value	YG (n=11)	MAG (n=11)	*p* value
FVC (L)	5.38±0.66	4.70±0.71	0.03	101.88±11.46	105.49±10.85	0.46
FEV1 (L)	4.55±0.44	3.50±0.45	<0.001	98.58±9.36	96.93±7.81	0.66
FEV1/FVC (%)	85.73±6.28	74.73±5.46	<0.001	-	-	-
IC (L)	3.42±0.32	3.38±0.65	0.84	83.10±10.32	96.70±15.88	0.03
ERV (L)	1.85±0.48	1.33±0.67	0.05	104.25±29.38	97.37±54.56	0.72
MVV (L/min)	195±19	165±30	0.01	108±11	111±16	0.56
**DR**	0.22±0.06	0.22±0.05	0.89	-	-	-

Unpaired Student’s t test or the Mann-Whitney rank sum test. BMI: body mass index. DR: dysanapsis ratio. ERV: expiratory reserve volume. FEV1: forced expiratory volume in 1 second. IC: inspiratory capacity. FVC: forced vital capacity. MAG: middle-aged group. MEP: maximal expiratory pressure. MIP: maximal inspiratory pressure. MVV: maximal voluntary ventilation. YG: young group. Predicted values for the Brazilian population by Neder et al. (17). Values are expressed as the mean±standard deviation or median (minimum-maximum). *p*<0.05.

**Table 2:** Cardiorespiratory parameters during cardiopulmonary tests using a cycle ergometer.

	YG (n=11)	MAG (n=11)	*p* value
Growth rate (W/min)	28 (23-30)	25 (20-30)	0.15
**GET**			
Power (W)	121 (95-154)	111 (67-163)	0.36
VO2 (mL/kg/min)	15 (14-20)	14 (9-22)	0.89
VCO2 (mL/min)	1289±236	1219±264	0.52
RER	0.98±0.07	0.98±0.07	1.00
**Peak**			
Power (W)	248±41	214±46	0.08
VO2 (mL/kg/min)	31±4	29±7	0.37
VCO2 (mL/min)	3310±800	2726±520	0.06
RER	1.27±0.12	1.22±0.11	0.30
VE (L)	87.37±26.22	75.17±23.14	0.26
**Ventilatory Efficiency**			
OUES	2679.71±511.16	2507.94±435.53	0.41
VE/VCO2 slope	24.44±5.12	25.14±3.30	0.71

Unpaired Student’s t test or Mann-Whitney rank t Test. MAG: middle-aged group. GET: gas exchange threshold. OUES: oxygen uptake efficiency slope. RER: respiratory exchange rate. VE: lung ventilation. VE/VCO2 slope: minute ventilation - carbon dioxide production slope. VO2: oxygen uptake. VCO2: carbon dioxide production. YG: young group. Values are expressed as the mean±standard deviation or median (minimum-maximum). *p*<0.05.

**Table 3:** Cardiorespiratory parameters during constant load tests using a cycle ergometer.

	YG		MAG		Effect of age	Effect of intensity
	75% GET	125% GET	75% GET	125% GET	*p* value	*p* value
VO2 peak (mL/kg.min)	19.0±1.7	28.2±3.1	18.2±5.1	29.0±8.6	0.984	<0.001
EFL (%)	0.0±0.0	0.0±0.0	7.8±14.6	28.9±20.8	<0.001	0.009
Power (W)	94±18	151±25	84±21	139±35	0.089	<0.001
IC (L)	3.7±0.4	3.7±0.4	3.4±0.6	3.6±0.7	0.266	0.674
FEFmax (L/min)	1.5±0.3	2.7±0.4	1.4±0.3	2.9±1.0	0.376	<0.001
FIFmax (L/min)	1.8±0.3	2.8±0.4	1.8±0.4	3.4±1.1	0.376	<0.001

Two-way ANOVA. EFL: expiratory flow limitation. FEFmax: maximal expiratory flow. FIFmax: maximal inspiratory flow. GET: gas exchange threshold. IC: inspiratory capacity. MAG: middle-aged group. VO2peak: peak oxygen uptake. YG: young group. Values are expressed as the mean±standard deviation. *p*<0.05.
